# Iron Metabolism and Muscle Aging: Where Ferritinophagy Meets Mitochondrial Quality Control

**DOI:** 10.3390/cells14090672

**Published:** 2025-05-03

**Authors:** Rosa Di Lorenzo, Emanuele Marzetti, Helio José Coelho-Junior, Riccardo Calvani, Vito Pesce, Francesco Landi, Christiaan Leeuwenburgh, Anna Picca

**Affiliations:** 1 Department of Biosciences, Biotechnologies, and Environment, Università degli Studi di Bari Aldo Moro, Via Edoardo Orabona 4, 70125 Bari, Italy; rosa.dilorenzo@uniba.it (R.D.L.); vito.pesce@uniba.it (V.P.); 2 Department of Geriatrics, Orthopedics and Rheumatology, Università Cattolica del Sacro Cuore, L.go F. Vito 1, 00168 Rome, Italy; riccardo.calvani@unicatt.it (R.C.); francesco.landi@unicatt.it (F.L.); 3 Fondazione Policlinico Universitario “Agostino Gemelli” IRCCS, L.go A. Gemelli 8, 00168 Rome, Italy; coelhojunior@hotmail.com.br (H.J.C.-J.); picca@lum.it (A.P.); 4 Department of Physiology and Aging, University of Florida, 2004 Mowry Road, Gainesville, FL 32611, USA; 5 Department of Medicine and Surgery, LUM University, Str. Statale 100, 70010 Casamassima, Italy

**Keywords:** autophagy, cytokine, endolysosomal system, hepcidin, inflammation, labile iron, mitophagy, physical performance, sarcopenia, transferrin

## Abstract

In older adults with reduced physical performance, an increase in the labile iron pool within skeletal muscle is observed. This accumulation is associated with an altered expression of mitochondrial quality control (MQC) markers and increased mitochondrial DNA damage, supporting the hypothesis that impaired MQC contributes to muscle dysfunction during aging. The autophagy–lysosome system plays a critical role in MQC by tagging and engulfing proteins and organelles for degradation in lysosomes. The endolysosomal system is also instrumental in transferrin recycling, which, in turn, regulates cellular iron uptake. In the neuromuscular system, the autophagy–lysosome system supports the structural integrity of neuromuscular junctions, and its dysfunction contributes to muscle atrophy. While MQC was thought to protect against iron-induced cell death, the discovery of ferroptosis, a form of iron-dependent cell death, has highlighted a complex interplay between MQC and iron-inflicted damage. Ferritinophagy, the autophagic degradation of ferritin, if overactivated, can induce ferroptosis. Alternatively, aging may impair ferritinophagy, leading to ferritin accumulation and the release of toxic labile iron under stress, exacerbating oxidative damage and cellular senescence. Physical activity supports muscle health also by preserving mitochondrial quantity and quality and enhancing bioenergetics. However, therapeutic strategies for preventing or reversing physical function decline in aging are still lacking due to the insufficient understanding of the underlying mechanisms. Unveiling how disruptions in iron homeostasis impact muscle quality in older adults may allow for the development of therapeutic strategies targeting iron handling to alleviate age-associated muscle decline.

## 1. Introduction

Among the biological pathways contributing to muscle aging and the associated decline in physical performance, alterations in mitochondrial quality and function, alongside structural and functional muscle remodeling, play a critical role [1,2]. Recent studies have uncovered a link between defective iron handling in muscle, mitochondrial dysfunction, and systemic inflammation in older adults [3,4]. The labile iron pool has been found to be elevated in the muscles of older individuals with low physical performance, a phenomenon that has been associated with dysregulation in the expression of mitochondrial quality control (MQC) markers and an increase in mitochondrial DNA (mtDNA) damage [3,4]. These findings highlight the significance of inefficient MQC as a factor contributing to muscle dysfunction during aging [1].

The autophagy–lysosome system plays a central role in MQC. This system facilitates the tagging, engulfment, and degradation of proteins and organelles through autophagosomes, which subsequently deliver these materials to lysosomes for breakdown. Preclinical research has shown that inhibition of the autophagy–lysosome system results in dysfunctional neuromuscular junctions, muscle atrophy, and weakness [5]. In addition, the accumulation of lipofuscin, a nondegradable lysosomal aggregate composed of polymerized lipid and protein residues, has been documented in the muscles of both aged mice and humans. This accumulation may be an indicator of lysosomal dysfunction and compromised cellular quality control processes. Thus, alterations in autophagy and mitophagy are believed to contribute to muscle atrophy and the decline in physical function that characterizes aging [6].

MQC was initially believed to prevent cell death induced by iron overload. However, the discovery of ferroptosis, a form of iron-dependent cell death resulting from the lipid peroxidation of cellular membranes, has introduced a new perspective on the role of MQC in regulating iron-induced cell death [7]. An integral component of this process is ferritinophagy, the autophagic degradation and recycling of ferritin [8], which, if overactivated, can lead to ferroptosis [9].

Physical activity conveys several beneficial effects on muscle health, including the preservation of mitochondrial mass, promotion of mitochondrial dynamics and autophagy, and support of mitochondrial bioenergetics [10]. Nevertheless, a significant gap remains in our understanding of the underlying mechanisms driving the loss of physical function with aging, which hampers the development of effective therapeutic interventions. Given the rapidly growing population of older adults, identifying therapeutic targets to mitigate age-related muscle decline has become an urgent priority. In this context, understanding the mechanisms whereby iron dyshomeostasis contributes to the deterioration of muscle quality in older adults could have major implications for the development of therapeutic strategies targeting iron handling. Such interventions may offer new avenues to alleviate age-related muscle decline, thereby improving the health and quality of life of aging populations.

## 2. Iron, Mitochondria, and Aging

### 2.1. Iron and Mitochondria

Iron plays essential physiological roles, including its involvement in heme biosynthesis for oxygen transport by hemoglobin and muscle oxygenation by myoglobin, as well as its incorporation into iron–sulfur (Fe–S) clusters in cytochromes and cofactors of the mitochondrial electron transport chain complexes [11]. Iron homeostasis is tightly regulated at the molecular, cellular, and systemic levels. Indeed, low iron levels impair erythropoiesis, leading to anemia, while excess labile iron promotes reactive oxygen species (ROS) generation, which can damage cellular structures and organs [12].

**Figure 1:**
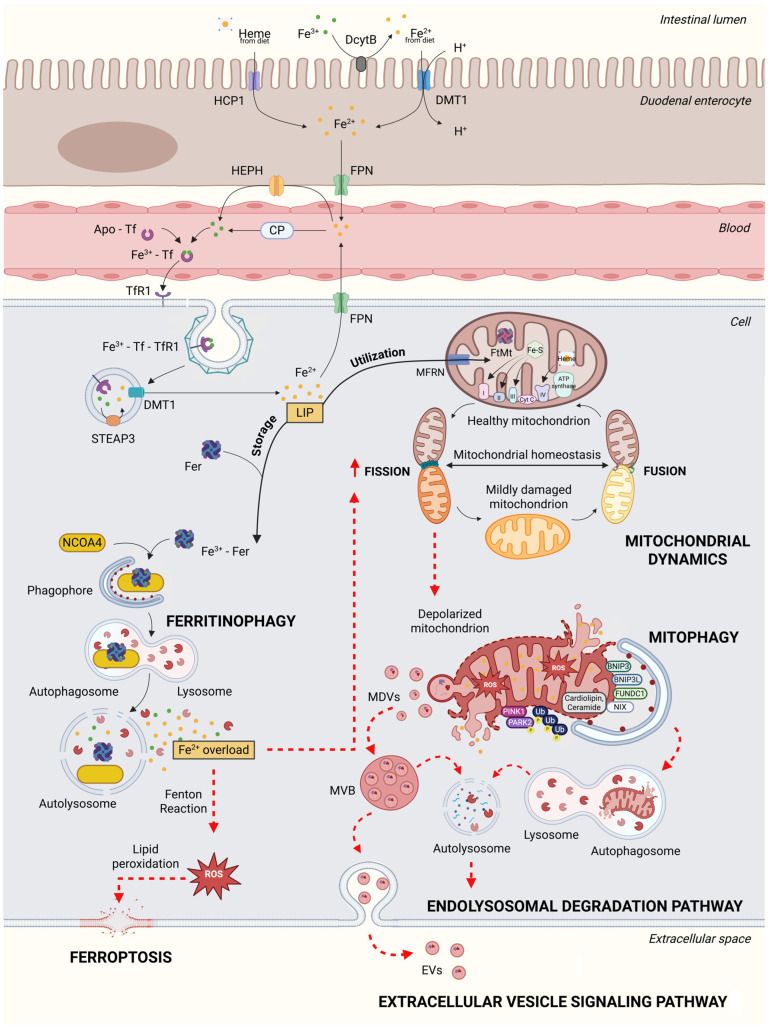
Mechanisms of iron transport and mitochondrial homeostasis. Dietary heme iron and ferrous iron (Fe^2+^) are absorbed by duodenal enterocytes and transferred into the bloodstream. Herein, iron binds Apo-transferrin (Apo-Tf), which allows for its mobilization. For cellular iron uptake, the ferric iron (Fe^3+^)–Tf complex binds to Tf receptor 1 (TfR1) at the plasma membrane and is internalized via a clathrin-coated vesicle. Iron released from Apo-Tf composes the cytoplasmic pool of labile iron that can be stored bound to ferritin (Fer) or can enter the mitochondria and form iron–sulfur (Fe–S) clusters and heme required for electron transport chain complexes. In response to low intracellular iron levels, nuclear receptor coactivator 4 (NCOA4) induces ferritinophagy, which can trigger Fe^2+^ overload and consequently reactive oxygen species (ROS) burst. The latter triggers ferroptosis, mitochondrial damage, and fission. To preserve mitochondrial homeostasis, damaged mitochondria, depending on damage severity, can undergo mitophagy and/or release mitochondria-derived vesicle (MDV) containing oxidized proteins and nucleic acids. In the cytosol, MDVs can form multivesicular bodies (MVBs) that can either pursue endolysosomal degradation or give rise to extracellular vesicles (EVs) released outside the cell. Red-dashed arrows highlight the pathways that are more relevant to iron dyshomeostasis and alterations in mitochondrial quality control. Abbreviations: BNIP3, BCL2-interacting protein 3; CP, ceruloplasmin; DcytB, duodenal cytochrome b; DMT1, divalent metal transporter 1; FPN, ferroportin; FtMt, mitochondrial ferritin; FUNDC1, FUN14 domain-containing protein 1; HCP1, heme carrier protein 1; HEPH, hephaestin; LIP, labile iron pool; MFRN, mitoferrin; NIX, NIP-3-like protein X; PINK1, PTEN-induced kinase 1; STEAP3, six-transmembrane epithelial antigen of prostate 3; Ub, ubiquitin. Created in https://BioRender.com (accessed on 24 April 2025).

Iron in the body exists in two distinct forms: heme iron (HI) and non-heme iron (NHI). HI is found at the center of a porphyrin ring in functional proteins such as hemoglobin, myoglobin, cytochromes, and Fe–S clusters [13,14] and is primarily derived from animal-based dietary sources such as meat and fish. In contrast, NHI, which constitutes approximately 30% of the bodily iron stores, is not associated with the porphyrin structure and is mainly found in plant-based foods [13,14]. Systemic iron uptake occurs in the duodenum, where enterocytes absorb dietary iron from the intestinal lumen. HI absorption is facilitated by the heme carrier protein 1 (HCP1), whereas NHI is taken up via the divalent metal transporter 1 (DMT1) [15] (Figure 1).

In addition, reticuloendothelial macrophages recycle iron from aged erythrocytes mostly in the spleen and release it into the bloodstream [16,17]. Here, iron is transported bound to transferrin (Tf), a glycoprotein synthesized by hepatocytes that possesses a high-affinity iron-binding site (Kd ≈ 10−20 M) [18,19,20,21]. The binding of iron to specific transporters is crucial for preventing oxidative damage to macromolecules, organelles, and cells.

Iron can exist in two states: the ferrous state (Fe^2+^), which is highly reactive and capable of reducing oxygen, and the ferric state (Fe^3+^). In conditions of iron overload, plasma Tf may become saturated, leading to an inability to fully bind free circulating iron [22]. In such circumstances, divalent metal transporters from the Zrt-Irt-like protein (ZIP) family intervene to absorb excess free iron [23]. The Fe^3+^–Tf complex enters cells through clathrin-mediated endocytosis upon Tf interaction with the transferrin receptor 1 (TfR1), a homodimeric glycoprotein. The binding of Fe^3+^–Tf to TfR1 is pH-dependent: at physiological pH (7.4), TfR1 binds Fe^3+^–Tf but not iron-free Tf, while the acidic environment of the endosome promotes the release of Fe^3+^, which is then reduced to Fe^2+^ by the ferrireductase six-transmembrane epithelial antigen of prostate 3 (STEAP3) and transferred to the cytoplasm via DMT1, coupled with H^+^ transport [24,25]. Cytoplasmic iron exists in a labile pool (~0.001 mM), typically bound to low-molecular weight molecules such as peptides, carboxylates, phosphates, or metallochaperone proteins [26,27]. The iron-free Tf–TfR1 complex is returned to the cell surface for recycling, enabling subsequent rounds of iron uptake (Figure 1).

The labile iron pool is regulated by two primary proteins: ferritin (Fer) and ferroportin (FPN). Ferritin, abundant in the bone marrow, spleen, and liver, is a multimeric protein composed of ferritin light (FTL) and heavy (FTH1) subunits that can sequester over 4000 Fe^3+^ ions in its core, utilizing the ferroxidase activity of its heavy chain to prevent oxidative damage [28,29]. The influx of labile iron into the cytoplasm induces the production of ferritin, highlighting the importance of storing and detoxifying excess iron to prevent cellular oxidative stress [30]. Iron is delivered to ferritin via poly (rC) binding protein 1 (PCBP1), which binds both RNA and iron at distinct sites [31] (Figure 1). Mice deficient in liver-specific PCBP1 exhibit oxidative stress, lipid peroxidation, and steatosis, highlighting the role of PCBP1 in mitigating cytoplasmic iron toxicity [32]. FPN, a secondary active transporter belonging to the major facilitator superfamily (MFS), is the only iron exporter responsible for transporting iron from cells into the circulation [33,34,35]. The levels of FPN are regulated by hepcidin, a defensin-like hormone that binds to FPN and induces its degradation [36,37]. Hepcidin expression is influenced by systemic iron levels and increases during iron overload while decreasing under conditions of anemia and hypoxia [38]. An increase in Tf also triggers the expression of hepcidin, which retains iron in macrophages and prevents iron absorption into enterocytes to avoid systemic iron overload [37,39] (Figure 1). Other molecular signals responsible for the transcriptional regulation of hepcidin are Tf saturation, erythropoietic activity, and inflammation [40]. Conversely, mutations in FPN are associated with hemochromatosis type 4, a disorder characterized by excessive iron accumulation [41]. Iron response element-binding proteins (IRP1 and IRP2) regulate the expression of ferritin and TfR1 at the transcriptional level by acting on the 3′ or 5′ untranslated region (UTR) of iron-responsive elements (IREs) [42,43]. IRP1 and IRP2 are homologous of the aconitase gene family and can sense cytosolic iron concentrations and respond by controlling the translation of Fer and FPN mRNA and the stability of TfR mRNA. Elevated iron levels reduce the affinity of IRP1 for IREs, leading to the conversion of IRP1 into its aconitase form. Conversely, when iron levels are low, IRP1 binds to the IRE with high affinity. IRP1 inhibits the translation of ferritin (Fer) mRNA when it binds to a single IRE located in the 5′ untranslated region (UTR) of the mRNA. On the other hand, when IRP1 binds to IREs in the 3′ UTR, it prevents mRNA cleavage and endonucleolytic degradation, thereby maintaining the transcription of TfR mRNA active [42]. Furthermore, a direct correlation has been observed in endothelial cells between the saturation of Tf-bound iron and FPN levels on the plasma membrane [44]. Once iron is transported outside the cell, Fe^2+^ is oxidized to Fe^3+^ by ceruloplasmin and/or hephaestin, mainly in enterocytes, and is subsequently bound to Tf for transport throughout the extracellular space [45].

Within cells, mitochondria are the main users and storage sites of iron. Herein, iron is incorporated into iron-containing proteins such as Fe–S cluster enzymes (e.g., electron transfer flavoproteins, NADH:ubiquinone oxidoreductase, succinate dehydrogenase subunits), heme-containing proteins (e.g., cytochrome bc1, cytochrome c, cytochrome c oxidase), and other iron-dependent enzymes [46] (Figure 1). While the abundance of iron-containing proteins in mitochondria highlights its critical role in mitochondrial function, the precise mechanisms by which labile iron reaches mitochondria from endosomes remain incompletely understood. One proposed model in reticulocytes suggests a “kiss-and-run” mechanism, where endosomes briefly interact with the outer mitochondrial membrane (OMM), facilitating direct iron transfer to mitochondria without the risk of generating reactive radicals in the cytosol [47]. In cardiac cells, iron is thought to enter mitochondria via fluid-phase endocytosis in a partially solvent-occluded manner [48]. In addition, studies in rat hepatocytes and yeast suggest that Fe^2+^, the primary form of iron in the cytosolic labile iron pool, can be imported into mitochondria driven by the mitochondrial membrane potential [49,50]. Iron must pass through both the OMM and the inner mitochondrial membrane (IMM) to reach the matrix, where most mitochondrial iron metabolism occurs. The import of Fe^2+^ into the intermembrane space may involve porins or a mitochondrial isoform of DMT1 (mDMT1) that has been identified in the OMM of HEK293 cells [51,52]. Subsequently, iron can be transported into the matrix through mitochondrial carrier family (MCF) proteins such as mitoferrin 1 (MFRN1), highly expressed in developing erythroid cells, and mitoferrin 2 (MFRN2), expressed ubiquitously, which are both located on the IMM [53,54]. Mitochondrial sideroflexins (SFXN), a family of transmembrane proteins [55], are also implicated in mitochondrial iron homeostasis [56], and their altered expression has been associated with various disorders, including mitochondriopathy or transient embryonic and neonatal sideroblastic anemia [46]. As heme synthesis occurs in mitochondria in close contact with the endoplasmic reticulum (ER), it is possible that heme is trafficked to the cytoplasm via the mitochondria–ER network, as shown in *Saccharomyces cerevisiae* [57]. In mammals, mitochondria–ER MCSs are referred to as mitochondria-associated membranes (MAMs). Mitochondria-derived vesicles (MDVs) may also play a role in heme trafficking [58]. MDVs can have either a single- or double-membrane structure, with a diameter ranging from 70 to 100 nm, and contain ligase proteins that are anchored to the mitochondria. These vesicles are specifically directed toward lysosomes and peroxisomes [59,60]. A key function of MDV-mediated heme “isolation” is to protect the cell from the harmful effects of free iron.

Iron storage within mitochondria is achieved through a specific mitochondrial ferritin (FtMt) (Figure 1), which is expressed in a tissue-specific manner in mammals (e.g., brain, in the medulla spinal cord, testes, heart, kidneys, and islets of Langerhans) [61,62]. Its main task is to sequester labile iron to prevent ROS formation [63]. Notably, FtMt overexpression compromises cellular iron homeostasis due to cytosolic iron depletion and mitochondrial iron overload [64].

### 2.2. Age-Related Mitochondrial and Iron Dysregulation

The role of iron in age-related oxidative damage and associated conditions has long been recognized, although the involvement of iron homeostasis in physiological aging warrants further exploration [65]. Both iron overload and deficiency have been observed in aging individuals and are associated with age-related disorders [66]. Iron accumulation in older adults has been observed particularly in liver diseases, kidney disorders, and Alzheimer’s disease [65,66]. Iron excess is particularly dangerous in senescent cells due to their inability to proliferate, which limits the redistribution and buffering of iron [67]. In these conditions, elevated levels of Fe^3+^–Fer and labile Fe^2+^ promote ROS-induced senescence [67]. On the other hand, age-associated iron deficiency has been linked to a reduced efficiency of dietary iron absorption and the sequestration of iron within macrophages, leading to decreased bioavailability for erythropoiesis [68].

In the skeletal muscle of aged rats, a high expression of Fer, increased iron transport via Tf, and a marked downregulation of TfR1 were observed, which were correlated with significant increases in NHI levels and oxidative damage to proteins and RNA [69,70,71]. NHI is a potent pro-oxidant metal that facilitates ROS generation, and its excess in muscle tissue has been associated with tissue atrophy [72,73]. NHI and total iron concentrations increase with age in skeletal muscle, plateauing in late middle age. While NHI continues to rise independent of Fer expression, a reduction in TfR1 has been linked to IRP2 downregulation [73].

Altered cellular iron transport and its accumulation within mitochondria have been associated with mitochondrial dysfunction [3,4,74,75]. Iron overload, mitochondrial dysfunction, and lipofuscin accumulation have been documented in liver cells [76]. The accumulation of lipofuscin with aging is observed in most postmitotic cells as non-disposable waste stuck in secondary lysosomes [77]. Lipofuscin has been identified as a store of redox-active iron, which can potentially lead to oxidative damage [77]. On the other hand, iron deficiency has been linked to reduced mitochondrial number and function in muscle cells.

Damaged mitochondria are cleared via mitophagy or proteasomal degradation; however, alternative mitochondrial recycling pathways via MDVs have been proposed [78,79,80]. During mitophagy, an autophagosomal membrane envelops the damaged mitochondrion, which is subsequently fused with a lysosome for degradation. Mitophagy can occur through two distinct pathways based on the dependence/independence on the PINK1/PARKIN axis [6]. The PINK1/PARKIN pathway is the best characterized route. Under basal conditions, PINK1 is cleaved and inactivated; however, mitochondrial stress or programmed mitophagy prevents PINK1 cleavage, leading to its accumulation on the mitochondrial surface, where it recruits PARKIN to ubiquitinate proteins for proteasomal degradation [6]. Other proteins, such as BCL2-interacting protein 3 (BNIP3), NIP-3-like protein X (NIX), and FUN14 domain-containing protein 1 (FUNDC1), facilitate PINK1/PARKIN-independent mitophagy, promoting mitophagosome formation. Various receptors, including BCL-2-like protein 13 (BCL2L13), FK506-binding protein 8 (FKPB8), Prohibitin 2 (PHB2), cardiolipins, autophagy and beclin 1 regulator 1 (AMBRA1), and neighbor of BRCA1 gene 1 protein (NBR1), also contribute to the execution of mitophagy [6]. In the context of iron deficiency in myotubes, mitochondria become surrounded by double-membrane structures, with mitochondrion–lysosomal co-localization and increased expression of mitophagy proteins [81].

Frataxin (FXN), a 17 kDa protein, plays a crucial role in mitochondrial iron homeostasis by acting as an iron chaperone during the synthesis of heme and Fe–S clusters. FXN also serves as an iron storage protein in the presence of excess metal, aids in the repair of oxidatively damaged Fe–S clusters in aconitase, and reduces ROS concentrations within mitochondria [82]. Indeed, the overexpression of FXN in *Drosophila* has been shown to counteract oxidative stress and extend lifespan [83]. Age-related iron imbalance impacts mitochondrial function, particularly in skeletal muscle, which serves as the body’s primary iron reservoir. Consistent with the findings in murine models, an increased expression of mitoferrin (MFRN) and reduced FXN levels have been observed in association with high labile Fe^2+^ concentrations in the skeletal muscle of older adults with low physical performance [4]. This suggests a potential dysregulation of mitochondrial iron handling, where MFRN upregulation may serve as a compensatory response to iron excess. However, FXN alone is insufficient to counteract iron overload, leaving free iron to compromise mitochondrial integrity via Fenton’s reaction [4]. Preclinical studies indicate that iron uptake by skeletal myocytes through TfR1 significantly decreases with aging, accompanied by the accumulation of intracellular labile iron [84]. Similar changes have been observed in the skeletal muscle of older adults, where TfR1 protein expression correlates with impaired physical function [3]. A potential mechanism underpinning these findings could involve TfR1 being diverted to a degradative pathway instead of being recycled back to the plasma membrane, leading to reduced receptor levels. Alternatively, TfR1 may be rerouted to the exosomal secretory pathway, a process that will be further discussed in the next section. Proper TfR1 recycling is critical for maintaining muscle physiology, as the inactivation of TfR1 impairs energy production in muscle cells, causing growth arrest and metabolic dysregulation, due to a failure to shift to fatty acid β-oxidation [85]. The link between muscle quality decline and disrupted TfR1 recycling warrants further investigation, and understanding the lysosomal degradation of TfR1 and its exosomal cargo could provide insight into iron mishandling and muscle function deterioration.

## 3. The Endolysosomal System and Ferritinophagy for the Regulation of Iron Metabolism: Converging Pathways?

Mitophagy plays a crucial role in maintaining cellular quality by selectively eliminating damaged mitochondria and regulating organelle numbers according to metabolic demands; however, excessive mitophagy can be detrimental to cells [86,87]. Excessive mitochondrial fission generates abundant mitochondrial debris, which activates mitophagy [88]. In contrast, when mitochondrial damage is mild, oxidized protein cargoes may be selectively incorporated into MDVs, which bud off from the mitochondria [59,60]. MDVs may thus serve as an alternative pathway to mitophagy and contribute to mitochondrial homeostasis [89]. These vesicles can follow one of two possible fates: (1) undergo endolysosomal degradation or (2) be secreted into the extracellular space as part of the many extracellular vesicles (EVs) [90,91,92,93]. The endosomal degradation pathway involves the internalization of molecules and fluids through membrane invaginations, which subsequently form vesicles or endosomes that fuse with lysosomes. Lysosomes contain hydrolytic enzymes that operate at acidic pH to degrade proteins, lipids, and carbohydrates. In contrast, EVs are lipid bilayer vesicles, typically ranging from 40 to 1000 nm in size, that can be released from any cell type. EVs play an essential role in cell-to-cell communication, facilitating the exchange of biomolecules such as proteins, nucleic acids, and lipids over long distances, and are involved in both physiological and pathological processes [78]. EVs can be categorized into exosomes (40–160 nm in diameter), which originate from the endosomal system, and microvesicles, which bud directly from the plasma membrane [94]. Exosomes are derived from intraluminal vesicles (ILVs) within multivesicular bodies (MVBs), which mature and either fuse with lysosomes for degradation or with the plasma membrane to release exosomes into the extracellular environment [95]. Recently, entire organelles or their fragments have been found to be transferred via MDVs, which may contribute to mitochondrial homeostasis [92,96,97,98,99].

MDVs are not dependent on the nuclear fission GTPase dynamin-related protein 1 (DRP1) and mainly originate from the OMM, although they can also arise from the IMM, thus partially including the mitochondrial matrix [91,100,101]. By delivering mildly depolarized mitochondria, MDVs can reduce cellular ROS levels, thereby contributing to preserving mitochondrial quality [102]. For example, stressed brown adipocytes release EVs containing oxidized and damaged mitochondrial fragments to maintain cellular homeostasis and support adaptive thermogenesis [103]. However, in conditions where mitophagy or the mitochondria–lysosome axis is impaired, cellular quality control is compromised, resulting in the accumulation of damaged mitochondria, misfolded proteins, and lipofuscin [104].

In older adults with physical frailty and sarcopenia (PF&S), the finding of elevated levels of EVs containing mitochondrial components suggests an attempt by the cells to eliminate dysfunctional organelles [98]. The involvement of MDVs in the intercellular transfer of mitochondria or mitochondrial components allows for an alternative explanation for such findings to be proposed. Accordingly, metabolically competent cells may release EVs containing functional organelles and/or mitochondrial constituents holding signaling roles to support the viability of cells with high levels of mitochondrial damage [100].

Cardiac myocytes begin eliminating MDVs even when mitochondria are only slightly oxidized, indicating that MDV formation may be independent of mitochondrial depolarization, autophagy signaling, and fission processes [105]. Cultured H9c2 myoblasts have been shown to continuously release MDVs [105]. In this system, damaged proteins resulting from local organelle dysfunction are removed under basal conditions without mitochondrial depolarization [60]. In contrast, mitochondrial stressors such as the mitochondrial inhibitor antimycin-A lead to an overproduction of MDVs [105]. Notably, the cargo of MDVs is determined by the target destination and the type of mitochondrial stress. A study by Todkar et al. [92] demonstrated that, depending on the stimulus, the same protein cargo can be directed either to EVs or to lysosomes. In cases of damaged mitochondria, most MDVs are directed to lysosomes, thereby blocking the alternative pathway of mitochondrial protein release into EVs. Conversely, the PINK1/PARKIN pathway promotes the inclusion of oxidized, damaged proteins into MDVs and inhibits the degradation pathway toward lysosomes. In the skeletal muscle, iron chelation in myotubes leads to reduced mitochondrial respiratory capacity, decreased mitochondrial protein levels, and a reduction in the mtDNA copy number, which supports the secretion of mitochondrial components through MDVs [81].

Recent studies have also suggested that the EV pathway may serve as an alternative mechanism to FPN for iron efflux, wherein MDVs facilitate the transfer of mitochondrial iron into EVs. This process allows cells with accumulated iron to clear excess metal and mitigate iron-induced damage. However, this occurs at the expense of recipient cells that absorb iron-rich EVs, potentially leading to oxidative damage and ferroptosis, a type of cell death caused by iron-dependent lipid peroxidation [106,107,108]. Ferroptosis is induced in satellite cells lacking TfR1, which impairs skeletal muscle regeneration [109]. Ferroptosis is closely associated with lysosomal activity, as lysosomes regulate iron metabolism by degrading ferritin and releasing free iron [110]. Lysosomal dysfunction or damage can result in the accumulation of lipid peroxidation, exacerbating ferroptosis [111]. EVs represent an alternative disposal system for damaged cellular components in the event of lysosomal dysfunction [112]. Indeed, the impairment of lysosomal activity leads to the accumulation of MVBs and increased EV release, with the subsequent overproduction of EVs when lysosomal activity is blocked [113]. Furthermore, iron-binding proteins such as Fer that leak from damaged lysosomes are loaded into EVs and released into the extracellular environment, thus augmenting the cellular iron pool [114]. Furthermore, the IRE–IRP system has been identified as a regulator of the EV marker protein cluster of differentiation (CD) 63. The IRE–IRP system is activated by iron accumulation and controls the transfer of Fer into CD63-positive EVs [115]. Other iron-related proteins, such as Tf and TfR1, have also been found in EVs, with their secretion being primarily induced by oxidative stress, which activates autophagy to dispose of intracellular iron [116,117]. EVs are also involved in iron redistribution [118]. Cytosolic iron export via FPN lowers intracellular iron levels by promoting the degradation of Fer through the proteasome [119]. However, Fer can also be degraded in lysosomes through ferritinophagy, a selective autophagic process that allows for the recycling of stored iron and the reduction in oxidative damage from iron overload [120]. When cellular iron availability is low, the corepressor of the nuclear receptor for the silencing transcription factor RE1 (REST) binds to the Fer gene promoter to induce its transcription [121]. Conversely, in the setting of iron excess, NCOA4 binds to Fer and triggers its degradation via the autophagy–lysosomal pathway. Ferritinophagy is considered a selective form of autophagy, in which excess intracellular Fer can induce iron-mediated apoptosis by activating NCOA4 [122]. Specifically, the Fer–NCOA4 complex localizes to the membrane of a proto-autophagosome and promotes the formation of a fully enclosed autophagosome, which fuses with the lysosome to degrade Fer and release iron into the cytoplasm for cellular use [122]. NCOA4 expression is upregulated during iron deficiency via hypoxia-inducible factor (HIF) and suppressed during iron excess through proteasomal degradation [123]. NCOA4-deficient mice accumulate Fer and iron in tissues at the expense of circulating iron [124]. Several factors modulate NCOA4 expression and ferritinophagy induction, including autophagy-associated genes (ATGs), which activate NCOA4 and promote intracellular iron transport and ferritinophagy, leading to ferroptosis [125,126]. Conversely, reducing NCOA4 or ATG expression mitigates iron overload and lipid peroxidation, thus protecting cells from ferroptosis [127]. NCOA4 expression can also be regulated through additional signaling pathways. The FTH1-NCOA4 complex binds to microtubule-associated protein 1A/1B-light chain 3- phosphatidylethanolamine conjugate (LC3II) in the lysosome, resulting in the release of Fe^2+^ and enhancement of the Fenton reaction that promotes ferritinophagy and ferroptosis [128]. The binding of NCOA4 to FTH1 can be inhibited by Yes-associated protein 1 (YAP1), a key regulator of the Hippo signaling pathway [129]. Finally, interleukin-6 (IL-6)/signal transducer and activator of transcription 3 (STAT3) and c-Jun N-terminal kinase (JNK)-transcription factor Jun (JUN) are additional regulators of NCOA4 expression [130,131].

## 4. Promoting Muscle Quality and Function and Iron Homeostasis: Research Gaps and Opportunities

Physical function plays a critical role in maintaining independence during late life. Older adults with functional limitations experience a more rapid decline in physical function compared to those with better physical performance [132,133,134]. However, the biological mechanisms underlying this accelerated decline in mobility-limited older adults are not well understood, and few strategies have proven effective in preventing mobility disability. Measures of physical performance, such as walking speed and chair-stand tests, are strong predictors of the incidence of mobility disability and all-cause mortality. In addition, muscle quality has emerged as a reliable indicator of functional capacity and mobility [1,135].

Iron metabolism in aging muscle has become an area of active research, as an improper handling of iron can lead to its accumulation in parenchymal tissues, negatively impacting muscle quality and function [4,72]. This accumulation may develop in the context of age-related chronic low-grade inflammation, wherein hepcidin signaling becomes upregulated, promoting cellular iron retention [40,136]. Elevated levels of muscle iron and plasma hepcidin have been observed in older individuals compared with younger controls and have been linked to reduced physical performance and increased levels of inflammatory markers such as C-reactive protein (CRP) and IL6 [1,3,4]. Within this pro-inflammatory environment, increased hepcidin expression may disrupt iron homeostasis by inhibiting the expression of the iron export protein FPN, leading to intracellular iron overload.

In addition, reduced mtDNA content and increased mtDNA damage have been observed in the muscle of older adults in association with dysregulated iron homeostasis and mitochondrial dysfunction [3]. Although multiple factors contribute to physical disability, substantial evidence suggests that mitochondrial dysfunction is a significant factor in functional decline [137,138,139]. Mitochondrial dysfunction is closely linked to the molecular pathways identified as hallmarks of aging [140,141]. Disruptions in cellular and mitochondrial iron transport have also been implicated in muscle aging, as they promote oxidative damage-mediated mutations and deletions, thereby reducing mitochondrial genome stability and impairing mitochondrial function. Moreover, ferroptosis has been proposed as a protective mechanism against age-related chronic damage [107].

A significant gap in knowledge is that most of the available evidence on the relationship among iron homeostasis, MQC, and ferroptosis originates from studies conducted in cell lines or tissues other than the muscle. Although these studies provide valuable mechanistic insights, their direct applicability to the skeletal muscle remains speculative. Indeed, the muscle possesses unique structural, metabolic, and regenerative properties, which may influence how these pathways are regulated or respond to stress and aging. However, preclinical studies indicate that age-related iron overload does affect functional parameters such as grip strength, muscle power, and muscle structural integrity [70,72,142]. Nevertheless, the trajectory of muscle quality in older adults and its associations with mitochondrial quality, lysosomal activity, iron metabolism, and measures of lower extremity tissue composition and physical performance remain insufficiently studied.

Preliminary evidence in humans suggests that elevated circulating hepcidin levels and dysregulated muscle iron in older adults with low physical performance are associated with reduced mitochondrial quality [1,3,4]. To advance this field, longitudinal studies examining the relationship between markers of iron regulation and mitochondrial dysfunction in older adults with varying levels of physical performance are needed. In such studies, low-functioning individuals should be compared with high-functioning peers to determine whether greater impairments in these markers are present in the former group. Based on the current literature, it is hypothesized that higher levels of hepcidin, along with increased muscle iron dysregulation and more pronounced mitochondrial dysfunction, may correlate with diminished muscle performance (e.g., slower walking speed). Under the best auspices, the longitudinal analysis of blood and skeletal muscle markers would indicate that an increased level of circulating hepcidin, greater muscle iron dysregulation, and elevated mitochondrial dysfunction in older adults with low physical performance may predict a faster decline in walking speed and physical function. If iron overload is determined to be causally linked to muscle dysfunction and declining physical performance, iron chelators could potentially be used to enhance myocyte viability. However, a major limitation of current chelation therapies is their lack of selectivity for specific organs or macromolecular structures. Once distributed within tissues, these agents indiscriminately chelate iron, restricting their use in older adults who are especially susceptible to iron-deficiency anemia [143]. Furthermore, in skeletal muscle, iron is essential for oxygen transport via myoglobin. Thus, there is an urgent need for targeted therapeutic strategies that selectively reduce labile iron while maintaining the bioavailable iron required for normal physiological function [84].

An overview of the major preclinical and human studies that explored the relationship between iron metabolism and markers of muscle homeostasis is provided in Table 1.

## 5. Conclusions

Impaired structural and functional muscle remodeling, along with altered mitochondrial quality and function, plays a central role in muscle aging. While the molecular mechanisms underlying these processes remain largely unclear, emerging evidence points to a link between altered muscle iron handling, mitochondrial dysfunction, and systemic inflammation. Understanding the mechanisms through which iron dyshomeostasis contributes to muscle quality decline in advanced age could have significant implications for developing therapeutic strategies that target iron handling to alleviate age-associated muscle decline.

## Figures and Tables

**Table 1:** List of the main studies investigating the relationship between iron metabolism and mitochondrial homeostasis in muscle.

Type of Study	Main Findings	Main Biochemical Measures	References
Preclinical	Elevated total iron in muscles of old rats and altered expression of genes and proteins involved in redox homeostasis and iron handling	Total iron quantification Gene expression assays (cDNA array and RT-PCR) 2-D proteomic analysis and mass spectrometry of 87 protein spots	Altun et al., 2007 [71]
Preclinical	Elevated non-heme iron content and RNA oxidative damage in muscles of old rats, especially following hindlimb suspension for two weeks	Nuclei acid oxidation Non-heme iron quantification	Hofer et al., 2008 [69]
Preclinical	Elevated non-heme iron content in muscles of old rats and altered expression of proteins involved in iron handling	Non-heme iron quantification Protein expression of TfR1, DMT1, IRP1, and ferritin subunits	Jung et al., 2008 [70]
Preclinical	Elevated non-heme iron content, mtRNA oxidation, and pro-apoptotic signaling in muscles of old rats	Non-heme iron quantification Nuclei acid oxidation Mitochondrial transition pore opening assay Caspase-3 and caspase-9 enzymatic activity	Seo et al., 2008 [75]
Preclinical	Elevated non-heme iron content and RNA oxidative damage in muscles of old rats, which were attenuated by lifelong moderate calorie restriction	Nuclei acid oxidation Non-heme iron quantification	Xu et al., 2008 [72]
Preclinical	Elevated non-heme iron content and macromolecular oxidative damage, altered expression of proteins involved in iron handling in muscles of old rats	Non-heme iron quantification Oxidative damage to nucleic acids, lipids, and proteins Gene and protein expression of TfR1, DMT1, ZIP14, ferroportin, hemojuvelin	Xu et al., 2012 [84]
Preclinical	Elevated non-heme iron content in muscles of old rats and altered expression of proteins involved in iron handling, with no effect of systemic administration of iron chelator	Non-heme iron quantification Gene and protein expression of TfR1, IRP1, IRP2, and ferritin light chain, ferroportin	DeRuisseau et al., 2013 [73]
Preclinical	Growth arrest and blunted energy production in muscles via fatty acid oxidation in mice with tissue-specific inactivation of TfR1	Targeted metabolomic and proteomic analysis Enzymatic activity of aconitase Protein expression of OXPHOS complexes and TfR1	Barrientos et al., 2015 [85]
Preclinical	Elevated non-heme iron content and macromolecular oxidative damage in muscles of rats injected with iron dextran	Non-heme iron quantification Protein expression of thioredoxin, TXNIP, catalase, SOD1, SOD2, GRX2, RyR1, Calstabin 1 Protein and lipid oxidation Enzymatic activity of GPx and SOD	Liang et al., 2019 [142]
Human	Elevated total iron content and mtDNA damage, altered expression of proteins involved in redox homeostasis and iron handling in muscles of physically inactive older adults	Total iron content Protein expression of OGG1, ZIP8, and ZIP14 Plasma quantification of ferritin, hepcidin, IL6, and CRP Oxidative damage to mtDNA	Picca et al., 2019 [3]
Human	Larger labile iron pool, altered expression of proteins involved in mitophagy and iron handling, increased mtDNA damage in muscles of physically inactive older adults	Quantification of labile iron pool Protein expression of LC3BII/I and p62 Quantification of mtDNA^4977^	Picca et al., 2020 [4]
Preclinical	Exposure to iron chelator increased mitochondria-derived vesicle secretion by myotubes knocked down for mitophagy-related proteins	mtDNA copy number Protein expression of BNIP3, BNIP3L, DNM1L, FUNDC1, LC3B, HSP70, Flotillin-1, OXPHOS complexes Mitochondria-derived vesicle isolation Enzymatic activity of citrate synthase and HADH Mitochondrial respirometry	Leermakers et al., 2020 [81]
Preclinical	Activation of ferroptosis and impaired skeletal muscle regeneration in satellite cell-specific TfR1 deletion	Non-heme and total iron quantification Gene and protein expression of ferritin, MyoD, NRF2, Pax7, PGC-1α, and transferrin	Ding et al., 2021 [109]

Abbreviations: BNIP3, Bcl-2 and adenovirus E1B 19-kDa-interacting protein 3; BNIP3L, Bcl-2/E1B 19 kDa-interacting protein 3-like protein; cDNA, complementary DNA; CRP, C-reactive protein; DMT1, divalent metal transporter 1; DNM1L, dynamin-1-like protein; FUNDC1, FUN14 domain-containing protein 1; GPx, glutathione peroxidase; GRX2, glutaredoxin 2; HADH, β-hydroxyacyl-CoA dehydrogenase; HSP70, heat shock protein 70; IL6, interleukin 6; IRP, iron regulatory protein; LC3B, microtubule-associated proteins 1A/1B light chain 3B; mtDNA, mitochondrial DNA; mtRNA, mitochondrial RNA; MyoD, myogenic differentiation 1; NRF2, nuclear respiratory factor 2; OGG1, 8-oxoguanine DNA glycosylase; OXPHOS, oxidative phosphorylation; Pax7, paired box domain 7; PGC-1α, peroxisome proliferator-activated receptor-gamma coactivator 1 alpha; RT-PCR, real-time polymerase chain reaction; RyR1, ryanodine receptor 1; SOD, superoxide dismutase; TfR1, transferrin receptor 1; TXNIP, thioredoxin-interacting protein; ZIP, Zrt/Irt-like protein.

## Abbreviations

The following abbreviations are used in this manuscript

AMBRA1	Autophagy and beclin 1 regulator 1
ATG	Autophagy-associated gene
BCL2L13	BCL-2-like protein 13
BNIP3	BCL2-interacting protein 3
CRP	C reactive protein
DMT1	Divalent metal transporter 1
ER	Endoplasmic reticulum
EV	Extracellular vesicle
Fe^2+^	Ferrous iron
Fe^3+^	Ferric iron
Fer	Ferritin
Fe–S cluster	Iron–sulfur cluster
FKPB8	FK506-binding protein 8
FPN	Ferroportin
FTH1	Ferritin heavy chain
FTL	Ferritin light chain
FtMt	Mitochondrial ferritin
FUNDC1	FUN14 domain-containing protein 1
FXN	Frataxin
HI	Heme iron
HIF	Hypoxia-inducible factor
IL-6	Interleukin-6
STAT3	Signal transducer and activator of transcription 3
ILVs	Intraluminal vesicles
IMM	Inner mitochondrial membrane
IREs	Iron-responsive elements
IRP1/2	Iron responsive element binding protein-1/2
JNK	c-Jun N-terminal kinase
JUN	Transcription factor Jun
LC3II	Microtubule-associated protein 1A/1B-light chain 3
LF	Low functioning
LIP	Labile iron pool
MAM	Mitochondria-associated membrane
MCF	Mitochondrial carrier family
MCS	Membrane contact sites
mDMT1	Mitochondrial divalent metal transporter 1
MDVs	Mitochondria-derived vesicles
MFRN	Mitoferrin
MFS	Major facilitator superfamily
MQC	Mitochondrial quality control
mtDNA	Mitochondrial DNA
MVB	Multivesicular body
NBR1	Neighbor of BRCA1 gene 1 protein
NCOA4	Nuclear receptor coactivator 4
NHI	Non-heme iron
NIX	NIP-3-like protein X
OMM	Outer mitochondrial membrane
PCBP1	Poly(rC) binding protein 1
PF&S	Physical frailty and sarcopenia
PHB2	Prohibitin 2
PINK1	PTEN-induced kinase 1
REST	Nuclear receptor of the silencing transcription factor RE1
ROS	Reactive oxygen species
SFXN	Sideroflexins
STEAP3	Six-transmembrane epithelial antigen of prostate 3
Tf	Transferrin
TfR1	Transferrin receptor 1
UTR	Untranslated region
YAP1	Yes-associated protein 1
ZIP	ZRT-IRT-like protein
